# Experiences of an HCV Patient engagement group: a seven-year journey

**DOI:** 10.1186/s40900-021-00249-2

**Published:** 2021-01-25

**Authors:** UNC Chapel Hill HCV Patient Engagement Group, Scott Kixmiller, Anquenette P. Sloan, Summer Wadsworth, Finton Brown, Lourdes Chaney, Larry Houston, Kim Thomas

**Affiliations:** 1 Patient Engagement Group Member and Co-Author, The University of North Carolina at Chapel Hill, Chapel Hill, NC USA; 2 University of Florida, Gainesville, FL USA; 3 The Johns Hopkins Hospital, Baltimore, MD USA

**Keywords:** Patient centered research institute (PCORI), Patient centered, Patient engagement group (PEG), Hepatitis C (HCV), Healthcare, Research studies, Drug trials, Insurance companies, Infectious diseases, Minority participation

## Abstract

Historically, few publications exist where patient engagement in clinical studies is a driving force in study design and implementation. The Patient Centered Outcomes Research Institute (PCORI), established in 2010, employed a new model of integrating stakeholder perspectives into healthcare research. This manuscript aims to share the experience of a Patient Engagement Group (PEG) that has engaged in hepatitis C (HCV) clinical research alongside investigators conducting two studies funded by PCORI and to inspire others to get more involved in research that can impact our healthcare and health policies.

There are many gaps in treating infectious diseases. Traditionally, treatment and research have been strictly clinical/medical approaches with little focus on the biopsychosocial aspects of individual patients. Our PEG reflected on its own personal experiences regarding how research design can affect study implementation by including patients who are normally excluded. We considered barriers to treatment, out of pocket costs, access to insurance, and patient race/ethnicity. Common themes were discovered, and four major topics were discussed. In addition, measures used in the two studies to collect patient data were considered, tested, and implemented by the group.

We describe in detail how we were formed and how we have worked together with researchers on two PCORI funded projects over the past 7 years. We formulated and implemented guidelines and responsibilities for operating as a PEG as well as appointing a chair, co-chair, and primary author of this manuscript.

Written from the perspective of a PEG whose members experienced HCV treatment and cure, we provide lessons learned, and implications for further research to include patients. PEGs like ours who are included as active partners in research can provide useful input to many areas including how patients are treated during clinical trials, how they interact with research teams, and how the clinical benefits of drugs or devices are defined and evaluated. PCORI believes engagement impacts research to be more patient-centered, useful, and trustworthy, and will ultimately lead to greater use and interest of research results by the patient and the broader healthcare community.

## Plain English summary

Few articles are published that describe patient engagement as a part of research from start to finish. The Patient Centered Outcomes Research Institute (PCORI) started a model of including patients into healthcare research. This manuscript shares the experience of a Patient Engagement Group (PEG) who has participated in two hepatitis C (HCV) clinical research studies funded by PCORI. Its aim is to inspire others to get more involved with clinical research.

We describe in detail how the PEG was formed and how we have worked together with researchers over the past 7 years. We created and applied guidelines and responsibilities for operating as a PEG. We describe the process of collaborating, writing, and editing this article.

Our PEG reflected on its own personal experiences regarding how research design can affect study operation by including patients who are normally excluded. Treatment barriers, out of pocket costs, access to insurance, and patient race/ethnicity were researched. Common themes were discovered, and four major topics were discussed. In addition, measures used in the two studies to collect patient data were considered, tested, and implemented by the group.

Like PCORI, we believe engagement impacts research to be more patient-centered, useful, and trustworthy, and will ultimately lead to greater use and interest of research results by the patient and the broader healthcare community. Written from the perspective of a PEG whose members experienced HCV treatment and cure, we provide lessons learned, and implications for further research to include patients.

## Background

Healthcare has been an ever-changing field created and highly influenced by medical professionals, the pharmaceutical industry, and insurance companies. As the field evolves and the quest for better, more effective, and cost-efficient care advances, input from patients is becoming more critical and commonplace. Relatedly, engaging patients as stakeholders in patient-centered outcomes research (PCOR), study design, patient care, and treatment decision-making can ultimately benefit patients, caregivers, medical professionals, and health policy over the long term [1]. Very little has been published in the scientific literature from the patients’ perspective related to conducting clinical research. Yet consumer (patient) input drives other industries, why not healthcare?

The purpose of this article is to share our collective experiences as patients in the United States who have engaged in clinical research alongside investigators conducting two US studies funded by The Patient Centered Outcomes Research Institute (PCORI). We share how the seven of us came together as people who were once afflicted with Hepatitis C (HCV), got treated and cured, then bonded together in a patient engagement group (herein referred to as the “HCV-PEG”) under the guidance of a principle investigator to work on two PCORI-funded projects. As of August 2020, the HCV-PEG has been together 7 years. We offer our experiences with the hopes of inspiring other patients, researchers, healthcare professionals, hospitals, insurance companies and pharmaceutical companies to work together to develop and implement relevant and meaningful patient-centered research studies that address patient and medical needs.

The HCV-PEG was created to partner with research teams, identify unmet patient needs, and develop studies consistent with the aims of PCORI. We have participated in two PCORI-funded studies: “The Patient-Reported Outcomes Project of HCV-TARGET (PROP UP) [2] from 2015 to 2018 and “THE PRIORITIZE STUDY: A Pragmatic, Randomized Study of Oral Regimens for Hepatitis C: Transforming Decision-Making for Patients, Providers, and Stakeholders” (Sulkowski, et al., unpublished/submitted/in preparation/forthcoming) from 2016 to present (contract ends in 2021). Although these dates reflect the award period of the research contracts, we were actively involved for 1–2 years prior to each award to participate in proposal writing and study design decisions that helped get these studies funded by providing patient input.

### The beginning of the HCV-PEG

In mid-2013, a researcher from The University of North Carolina at Chapel Hill (UNC) School of Medicine’s liver program worked to create the HCV-PEG to assist with developing a study protocol and to submit a proposal to PCORI to evaluate patients’ experiences with undergoing different types of medical treatment for chronic HCV in order to better address mandatory criteria used by PCORI grant reviewers, such as patient engagement and patient-centeredness. In deciding how to build the HCV-PEG, the investigator chose from patients who had previous interactions with providers in the clinical setting and were felt to be representative of the types of patients being treated for HCV in 2013. Dr. Donna Evon, the lead investigator, explained that in 2013 when she began to write the proposal for PCORI, no guidelines were available on how to engage patients in research proposals and studies, so she needed to make decisions on how best to bring the patients’ perspective to bear on many study design decisions. She had previously conducted a qualitative study of patients to understand the types of information patients wanted to help them make treatment decisions about HCV medications [3]. One of the first tasks she asked of the members was to help with prioritizing patients’ informational needs; these would later translate directly into study outcomes. Dr. Evon added that until that time, most investigators did not think of asking patients to participate in study design and decisions but PCORI changed all that. “If an investigator wants to obtain funding from PCORI, they must have significant patient collaboration and input. You can also obtain other ‘stakeholder’ contribution like family members, nurses, and insurance companies. For me, I wanted patients to tell me what we needed to study and how to do it” [4].

### Our membership and Mission

Dr. Evon had a vision for the team of individuals she would ultimately invite to serve on the UNC HCV-PEG. It would be a diverse group of people at different stages in the treatment process (before or during treatment or cured). We were all contacted and asked to make a multi-year commitment in the summer of 2013. At that time, only two of the members had completed HCV treatment and were cured. Most of the members underwent one or more treatments for HCV in the past, and a few members received treatment with the new direct acting antiviral (DAA) drugs while serving as members of the HCV-PEG. This was fortunate as some members brought intimate knowledge of treatment options, discussions with doctors, symptoms and treatment side effects while serving on the HCV-PEG. We are thrilled to report that at the time of this writing, we have all been cured of HCV.

The membership of the HCV-PEG has remained relatively consistent over the past 7 years. We were originally a group of seven from UNC, but one member died in year two, and one member resigned in year six, leaving us with five original members. When we entered into a contract with a second PCORI project in 2016, we added two additional members bringing our total back to seven with ages ranging from 40 to 70, three men, four women, three African American, three Caucasian, and one Hispanic. Our members have brought unique and insightful perspectives to the research and have substantially strengthened the quality of the HCV-PEG. Personal details written by each member can be seen in Table 1.

**Table 1:** PEG Member Biographies

**Irvin “Finton” Brown (FB)**	
I was diagnosed with HCV in 1998 by my primary care physician. I do not know how I contracted the virus. I can thank my doctor for suggesting that I should get tested since I am a “boomer.” I had a liver biopsy in 2000 and it showed that there was virtually no liver damage. These tests were done at the University of Nebraska Infectious Diseases Clinic since I was living in Omaha. My doctor decided that the treatment at the time was not worth the effort since I am a mixed-race male part being African. He advised that I should wait and monitor my condition over the years. I moved back to NC in 2010. Here I got a new doctor at the UNC liver clinic. I was again monitored until a treatment was acceptable to my doctor. I took HARVONI for 3 months and have been HCV free ever since. While I was being tested at UNC Liver clinic, I got a call from Donna (Evon) at UNC. She asked if I would join a group of others who had also had experiences with HCV. I said yes and the UNC HCV-PEG was formed to help with PROP-UP. Then UF asked if we would help with PRIORITIZE. So here we are today.	
**Lourdes Chaney (LC)**	
I was born in Puerto Rico. My journey with HCV began in 1991 when pre-testing for surgery and test results showed that I was positive with the virus. In 1993, with minimal understanding of the disease, I was given my first treatment with Interferon/Ribavirin. After 2 months of treatment, a high white blood cell count kept me from continuing the treatment. For the next 20 years I attempted two more treatments spaced years apart but still had detectable virus. Finally, in 2014, Sovaldi was approved and a few months later, I started treatment with Sovaldi/Ribavirin and was cleared after 12 weeks. I joined the UNC HCV-PEG in 2014 and continue to be an active member. I am currently a Staff assistant for the local government in Florida with a degree in Business and working on bachelor’s degree in Computer Technology.	
**Alan Franciscus (AF)**	
In 1966 I began traveling throughout the United States doing odd jobs including working in a warehouse, string factory, civil service, highway worker, and retail before settling down and earning a work degree in computers and accounting. Subsequently, I began work as an accounting clerk in two organizations in the San Francisco Bay Area. During this time, I went back to college and earned an associate degree and was applying to college when I was diagnosed with hepatitis C (HCV).	
In 1989 I was diagnosed with HCV genotype 1. Shortly after my diagnosis, I was treated three times before being cured – daily injections of interferon (Intron-A) for 6 months; a high dose of daily injections of interferon (Infergen) for 10 months; a weekly injection of interferon (Pegasys) for 70 weeks.	
After my diagnosis, I found that there was a lack of information about HCV available to patients and their medical providers. As a result, I decided to start the Hepatitis C Support Project (HCSP), an HCV Advocate Website, and a national training program to help educate and support people living with hepatitis C, their medical providers, and other advocates.	
In 2013 I joined the UNC HCV-PEG. In 2019 I retired from UNC HCV-PEG, HCSP and HCV Advocate Website due to health concerns and did not participate in authoring this manuscript. I will cherish the memories and always be proud of the many people that were helped by my efforts.	
**Larry Huston (LH)**	
I am from a small town in North Carolina. I graduated high school in 1968 and East Carolina University in 1976. After graduation I moved to Richmond, Virginia where I got married, got divorced and used drugs to numb the disappointment. Despite myself, I worked hard and started a career selling merchandise to major retailers on the East Coast. As my business grew so did my drug use.	
Love found me again and was married in 1990. I became a father and responsible for a family of five. Drugs became my escape.	
On a routine physical, I was diagnosed with Hepatitis C, and my doctor gave me a choice: wait for the new drug options coming or go ahead with the current treatment plan. For the next 48 weeks I took Interferon, Ribavirin, and Telaprevir, and was cured.	
During my treatment I met Donna Evon. She was my treatment “shrink.” She asked me if I would review a binder that she was putting together to help educate people on HCV. I was happy to help. I learned so much about HCV, most of which I was clueless. Donna was also establishing a PEG study and invited me to participate. In the meantime, I was reinfected and treated with Epclusa and cured again. 7 years later, I am still here.	
My journey has been rewarding. I have learned so much about the HCV virus and treatment while meeting outstanding physicians and nurses. I have had a chance to work with my peers - free of judgment or prejudice. Today, I feel grateful and blessed to have been able to participate in such an important study.	
**Scott Kixmiller (SK)**	
My HCV journey began in 1998. While in treatment for Substance Use Disorder, I tested positive for HCV. I am certain I contracted it through IV drug use sometime between 1996 and 1998. I was afraid of what HCV was, but I spoke with a nurse at that treatment center who had knowledge of HCV and she provided me with a referral for further testing. In 1999, while seeing my gastroenterologist and my viral load had increased to over 9 million, I was invited to take part in a drug study using Interferon and Ribavirin. At the time, I was participating in 12-step groups and had several people who had experience with HCV treatment try to discourage me from getting treated with the drugs. They kept telling me that I would get depressed and that the medications would not work (at the time, one in ten successfully had sustained virologic response - SVR). I remember praying about it and one day, when I got home from work, I found in my mail an acceptance letter from Schering-Plough that said all my medications would be covered for the entire study.	
I cleared my HCV at 12 weeks but continued to administer medication and remained in the study protocol for a total of 44 weeks. I remember giving myself a shot of what felt like a mild flu every day at first, then three times a week. I used my support network to help get me through the tough times. Since clearing, I have been tested periodically through the years and remain with SVR.	
Nine years later, while pursuing a Master of Social Work degree, I found a position as a research assistant on one of Dr. Evon’s studies entitled, “A Randomized Controlled Trial of an Integrated Care Intervention to Increase Eligibility for Chronic Hepatitis C Treatment” [5]. I graduated in 2010 and became an addiction and mental health therapist as a Licensed Clinical Social Worker and Licensed Clinical Addictions Specialist in the state of North Carolina. Two years after the publication of Dr. Evon’s paper, I was invited to join the UNC HCV-PEG in August 2013. My experience as a master’s student enabled me to be able to volunteer as the lead author of our paper starting in early 2019.	
**Anquenette P. Sloan (AS)**	
I am what you may call a “military brat” born into a Marine Corps family living on Navy and Marine Corp bases until age 18 when I left to attend college. If you have spent any time around the military you know once a “brat” always, a “brat.” In the following 16 years I worked to finish my degree in Public Health working as a Personal Banker for a Record Company, as a Personnel Manager, and a Pizza Store Owner. I was married twice, had a blended family of 10 children: yours, mine, ours and someone else’s. Today, I am the proud grandmother of 15. My career in Public Health started at the height of the HIV/AIDS epidemic in the late 1980’s while working in the state of Ohio. I worked with the American Red Cross, the CDC, and the State Health Department of Ohio. For 28 years, I helped develop education programs and services for people living with HIV/AIDS and prevention programs as a Public Health Administrator Specializing in Infectious Diseases. I was appointed by the Governor to the State of Ohio Medical Board and served for 7 years.	
My journey with HCV began unbeknownst to me in 1990 when I received blood products during a five-hour surgery. I never thought about HCV but during the years that followed I had fatigue, loss of appetite, and fever, which can be written off as anything. My life was so busy I never thought that I could have a disease and of course we were all tested many times for HIV because of the proximity to infected people. In 2008, I retired and moved back to North Carolina. In 2011, I had a life-threatening accident that caused brain damage and loss of some motor skills on my left side. I received the diagnosis of being positive for Hepatitis C while in the hospital. I was being treated at UNC and was referred to Dr. Donna Evon in 2012, where she and the team recommended that I wait for new medications to be made available before I received treatment. Dr. Evon invited me to join the UNC-HVC-PEG in 2013. I began treatment at the UNC Gastrointestinal Clinic through a medication assistance program in 2014. I used Harvoni for 12 weeks in 2015, cleared the virus, and remain virus-free. I serve as a Co-Chair of the PEG and a member of the Steering Committee.	
**Kim Thomas (KT)**	
For most of my adult life, I was dealing with a lengthy period of physical or medical problems that stemmed from an auto immune disease (HIV), which I contracted from my male partner of many years. Once getting out of denial and getting my health in alliance with a regimen of medications, I became undetectable and have been for over 20 years.	
In between this time, I tested positive for HCV. Because I was already under a provider’s care, I was offered treatment under a research study that used a combination of daily Pegylated Interferon injections with twice a day Ribavirin medication. The study/treatment regimen was supposed to last 48 weeks, but because I did not respond to treatment, it was discontinued.	
Later in 2013–2014, another new drug, Harvoni was introduced that my insurance paid for, so I began a 24-week regimen. It was successful and I was cured of HCV. Also, in 2013, my providers chose me to share my experiences and be a part of a randomized controlled trial which is an Integrated Care Intervention that is to increase the Eligibility for Chronic Hepatitis C treatment. I joined the UNC HCV-Patient Engagement Group and continue to be a regular contributor.	
I have worked, volunteered, and retired at clinics and psychiatric hospitals. I started my career with a bachelor’s degree in Organizational Psychology Development with a concentration in Dual Diagnosis Disorders.	
**Summer Wadsworth (SW)**	
In the early-1980’s after severe flu-like symptoms, I was diagnosed with non-a/non-b Hepatitis now known as Hepatitis C or HCV. At the time, there was not much that was understood about HCV nor was there a treatment. I lived life with chronic pain, fatigue and was treated for depression. Still over the decades, I participated in local theatre, graduated college, got married a couple of times, had a daughter who is now in college, and taught middle and high-school theatre. Several years ago, I participated in an HCV clinical research trial but became anemic, had viral breakthrough, and had to discontinue the trial. Following a year post treatment failure, I decided to try again using standard of care treatment which included not only Interferon and Ribavirin but also Solvaldi. Within 12 weeks there were no levels of virus detected and I was considered cured. I used to like to say that I was living “harmoniously” with my hepatitis, but the truth is I really did not understand how sick I was until I wasn’t anymore. I joined the UNC HCV-PEG in August 2013 and am currently a Co-Chair representing the PEG and am a member of the Executive Committee.	

As the HCV-PEG has grown so has its original mission that was formulated at the outset. “The mission of the UNC HCV Patient Engagement Group (HCV-PEG) is to integrate patient involvement and feedback into the complete research cycle of study development, study conduct, and dissemination of study findings, in order to advance Hep C research that is patient-centered, that is, consider the patient’s perspective as a critical aspect of conducting patient-centered outcomes research (PCOR). HCV-PEG members are viewed as *patient partners* and hired as *consultants/vendors* on research projects, a distinctly separate relationship from receiving care as a patient at UNC or participating in a research study as a study” [6].

Our membership, tasks as individuals within our group, and our designated representatives to the larger research bodies have been consistent, but fluid over the years. We have carved out a niche in terms of what we have contributed. Commonly in the investigators’ bi-annual Methodology Reports submitted to PCORI, [7] the work of the HCV-PEG is highlighted and implemented by the HCV-PEG:

1. Defining research topics and formulating study questions
2. Identifying a study population and choosing interventions, comparators, and outcomes
3. Developing optimal strategies for recruitment and retention of study participants
4. Conducting a study and analyzing results
5. Disseminating research findings into clinical practice

Although these activities are all relevant to major multicenter research projects, the governance for these projects can be quite complex, typically including a coordinating center, multiple clinical sites, a Steering Committee, an Executive Committee, a data and safety monitoring board, contractors, and often a sponsor oversight body [7]. How the HCV-PEG would fit into these organizational structures, interact with the researchers and other staff members, and how to meaningfully contribute to the research project – these were all questions we had to figure out.

### Responsibilities, guidelines and compensation

As the HCV-PEG was developed to serve on various projects, specific goals, responsibilities, and guidelines [6] were formulated to guide our engagement. During the grant-writing and conduction of the first PCORI study, the lead investigator presented the PEG with expectations of involvement and areas in which she needed patient input on study design features. See Table 2 for initial set of HCV-PEG responsibilities.

**Table 2:** Responsibilities (“Scope of Work”) of the HCV-PEG [6]

• Attend and actively participate in up to 6 meetings per year	
• Share perspective of patient or research participant	
• Secure access to computer and printer in your local area in order to review/edit study documents	
• Review study documents, providing suggestions and feedback	
• Assist investigators develop study design features	
• Select and finalize patient surveys and measures	
• Test the web-based data capture system and other elements of data collection	
• Participate in conference calls when requested	
• Review ongoing study progress	
• Provide feedback on how to modify and improve the research plan	
• Offer solutions to solve recruitment, consent, enrollment, retention challenges	
• Identify mediums for the dissemination of study findings to the lay public such as social media outlets, community engagement, and patient advocacy organizations	
• Provide feedback on data analysis, interpretation, and scholarly work (abstracts, papers, presentations)	
• Ensure project meets enrollment and completion timelines and study milestones	
• Attend Kick-Off meeting and host a Patient Panel to help investigators learn about the patients’ perspective	
• Participate in online training to understand basic clinical research (CITI training [8])	
• Review and approve Meeting Minutes	
**Co-Chairs' Additional Responsibilities **	
• Attend meetings and conference calls	
• Assist with drafting, editing, seeking approval, and distribution of PEG Meeting Minutes to other members and the Steering Committee	
• Work with investigators to draft documents requested by PCORI for progress reports and final reports	
• Serve as liaisons between the HCV-PEG and Steering Committee	
• Foster a reciprocal relationship, bi-directional communication, and sharing of information between the larger HCV-PEG and the investigators	
• Bring the collective views of the larger HCV-PEG to the investigators	
• Provide updates to the HCV-PEG on investigator and Executive Committee decisions, study progress and challenges	

The following reflects the expansion of the guidelines experienced by our PEG which could be beneficial in establishing future patient engagement groups.

- PEG Co-Chairs should be identified. On top of regular group member requirements, the Co-Chairs agreed to additional responsibilities and may be assigned as sitting members on the project committee such as the Steering Committee, Analyses Committee and Executive Committee. As members of these trans-disciplinary study committees and liaisons with the PEG and respective committees, the Co-Chairs are responsible for fostering a reciprocal relationship, bi-directional communication, and sharing of information between the larger PEG and the study investigators. Lastly, the Co-Chairs present the views of the larger PEG to the investigators and provide updates to the PEG on investigator and Committee decisions, study progress and challenges. Direct representation on these higher-level study committees ensured patients are collaborators in decision-making and accountability within these projects.
- Discuss project activities during one or two-hour face to face meetings over dinner, sometimes over phone/email, make decisions based on PEG feedback and then include decisions in various research and grant proposals.
- Establish guidelines so that all members and study stakeholders have a consistent and clear understanding of expectations. Any amendments to the guidelines should be provided in writing and signed in agreement.
- A “Kick-off” meeting should be held for the investigators to learn more about the PEG and the patient perspective. PEG members should complete the Collaborative Institutional Training Initiative (CITI Program) training to understand research with human subjects. “CITI is dedicated to promoting the public’s trust in the research enterprise by providing high quality, peer-reviewed, web-based educational courses in research, ethics, regulatory oversight, responsible conduct of research, research administration, and other topics pertinent to the interests of member organizations and individual learners.” [8].
- Establish a consultant/vendor agreement whereby PEG members are compensated for service and time. Effective patient engagement is a time-consuming process and can be a significant investment [9]. PEG members have been considered patient partners on research projects and are consultants whose agreements are renewed annually. Compesation rates have varied based on the project but may range from 50-125 per hour for travel to and from meetings, time spent in meetings, and review/drafting of study-related documents when needed. Depending on the project, a compensation check was mailed to our homes with 2-4 weeks or a direct deposit was made within 2 weeks after submission of a timesheet. In this consulting capacity, we were not considered 'research participants' therefore, federal, state, and university healthcare regulations related to the privacy and Health Insurance Portability and Accountability Act (HIPAA) did not apply to this consulting relationship.
- Depending on the project, PEG meetings may be organized and facilitated by either the Principal Investigator, Project Manager and/or our two Co-Chairs. Members should attend > 80% of scheduled meetings, arrive on time, and stay for the full duration of the meetings.
- Research staff may provide clerical support, facilitate communication via email or phone, and provide refreshments/food for face-to-face meetings they have planned. Meetings planned by the PEG depend on each member having an email, phone or access to a computer, and printer to review and edit study documents. Expect to review study documents while providing feedback to study investigators to design study features. Other tasks may include selecting and finalizing patient surveys and measures while testing the web-based data system and other elements of data collection.
- PEG members are expected to exhibit appropriate and professional conduct, respect for others’ opinions, and always use respectful language. We as members are asked to respect the privacy and personal information shared.
- PEG participation should be acknowledged on all scholarly works stemming from projects/ studies which includes any oral presentations or scientific publications by the investigators.
- Due to the public nature of these projects and roles, members may be mentioned by name, therefore, a written agreement should be made including the choice to opt-out in cases where anonymity may be important.
- Members should be encouraged to offer solutions on how to solve recruitment, consent, enrollment, and patient retention challenges. PEG may recommend, and develop, a multi-tiered system of centralized patient-outreach to study participants to ameliorate the study challenges. Members are expected to help identify ways to disseminate study findings to the public especially through social media, community engagement, and patient advocacy groups, provide feedback on data analysis, and scholarly work (abstracts, papers, presentations), and help ensure that projects meet enrollment and completion timelines as well as study milestones.

### The HCV-PEG experience comes together

The patient engagement group organized to form a mutual understanding of why we were chosen to participate. As we learned and actively participated in the process ourselves, we came to a unanimous conclusion that there have been many gaps in developing and treating patients with infectious diseases without including the patients’ perspective in what is studied or how it is studied. These patient perspective gaps exist in nearly all disease states so, it is not just a ‘one size fits all’ method. Historically, treatment and research have been strictly clinical/medical approaches with little focus on the biopsychosocial aspects of individual patients nor tailored to the respective disease impact on patients’ objectives, needs and priorities.

We asked ourselves how we can affect research to include patients who have not been normally considered for treatment. We remembered having spirited discussions with a lot of great ideas including how to address patients’ barriers to treatment like transportation to and from the numerous doctor’s appointment and labs, and how many out-of-pocket co-pays are needed for each appointment, and patients that do not have insurance. Additional consideration was given to African American and other minority communities’ historical mistrust of doctors and use of medications as seen in the Tuskegee Experiments [10]. We also added that many members were informed of an HCV diagnosis without sensitivity and empathy as well as without any kind of comprehensive information we could take to our families and friends about our new HCV diagnosis. We wanted to express to researchers and doctors that these things matter and should be incorporated into standardized care. Other questions that spurred a great deal of emotion and flashback to our own treatments included:

- How to address skills in coping with treatment? Who can the patient turn to?
- How are people paying for and receiving medication over the course of weeks/months?
- How to communicate with providers during treatment? What to ask?
- How to make an informed decision about whether to proceed with treatment.
- Are there any co-morbidities to address? Does the physician know how to handle them?
- How did we decide which “measures” (side effects during and after treatment, etc.) to use for patients participating in the studies? What was that process like for us as a PEG?
- What was a reasonable amount of time for study participants to complete questionnaires?
  How did we decide this?
- What was our process like deciding on the Confidentiality Statement, mission statement, and consulting agreements?

It was not easy deciding, but the group gave serious and careful thought of our own experiences as we combed through meeting minutes dating back to 2014 and decided on the top four “topics” we will address.

A common theme amongst the HCV-PEG members is that we had our own HCV treatment experiences and felt like many other patients could relate to similar encounters. Most of us, if not all, had to wait for treatment after we were told we had HCV and decided that pursuing medication was a good idea. A pertinent question and topic, ‘How do/did you communicate with providers during treatment?’ was formulated and thought to be valued enough for everyone to consider, especially when designing research and treatment protocols. Having effective communication measures in place for doctors and patients can greatly affect treatment outcomes.

What we found was that we had to be persistent in communicating with doctors and other providers that we wanted to treat our HCV, even if months had passed since the decision to start medication was made. Some struggled with delays in getting treatment because improved HCV medications were on the horizon and were projected to have less side effects and faster cure rates. Sometimes this meant that we had to make consistent efforts to get treatment started and it was our responsibility to keep up with it by communicating with doctors about having a clear intention of pursuing medication. Others had to express their fears with doctors because they had already failed previous treatments which had unpleasant side-effects but trudged through anyway. With this stated, based on our collective experience, we recommend that patients, researchers, and providers have a clear line of communication with everyone so that any fears as well as responsibilities can be addressed before beginning treatment.

Along with effective communication, having a clear understanding of “how are patients paying for and receiving their treatments and medications” is another topic we thought to be of value to providers and researchers. This includes making sure patients keep up with insurance, enrolling in drug coverage, communicating with the pharmacy, and knowing when medication is about to run out. As many know, just because the doctor recommends treatment and prescribes medication doesn’t mean that the insurance company will authorize it. The process of getting prior authorizations and filing claims itself can be enough to discourage patients and providers. Having a thorough understanding of navigating the insurance minefield is vital in whether a patient will be able to start, let alone complete the course of treatment.

Another helpful question asked by PEG members was, “How do you consistently keep patients engaged through the course of treatment?” Meetings between patients and providers, especially during potentially tough times of depression, physical side effects, and not having doctor visits for months at a time, are very helpful in aiding successful treatment outcomes. Phone calls and email newsletters that communicate study progress helped active study participants feel like they are kept in the loop with researchers, not on the outside looking in. Members also agreed that having a robust orientation to treatment including the importance of being an active study participant and that being involved with research will help others afflicted with HCV. Just knowing that as a study participant, patient input will contribute to HCV literature and can enable feelings of success and purpose. We agreed that having this psychological boost can enable confidence during tough times as well as increase intrinsic motivation to be compliant with all levels of treatment and study protocols. Feeling valued is important. In addition to altruism, patients who were compensated for their time outside of regular clinic visits kept them engaged and invested in not only their own treatment but in the study integrity.

Next, we had to decide “Which measures were used for the programs?” (social, occupational, mental and physical). When contemplating which methods to use in studies, it helped that each PEG member had his or her own experiences in treatment especially with respect to medication side effects. We were presented many different sets of questions and scales that had to be narrowed to what the best and most efficient way might be to collect the data. Each member had a chance to weigh in and a consensus was made on what types of questions to include for patients who were actively involved in a drug study. Members were also asked to complete the sets of questions and to time themselves. The results were brought back to the group so that more editing could be done. This was determined valuable as many patients in studies might get bored or refuse to answer questions all together if it was determined to take too much time. Future researchers should involve patients at this level if they want quality and consistent engagement in their studies.

### How can we contribute to the literature?

As participation in PRIORITIZE began to wind down, we asked ourselves “How else can we contribute?” At the time we began contemplating writing, almost 6 years of participation in the development and implementation of two large research studies have given us ample experience as members of a patient engagement group. It was suggested that we might want to make a group contribution to the new science of “Patient Engagement”. The body of scientific literature on patient engagement is still in its infancy, so we decided to embark on this article. One of the PEG members with a master’s degree in social work and writing experience was nominated to serve as the first author and spearhead this experiential article. Preliminary discussions for the article included how and when we would share information. Since we were embarking on this work ourselves, none of the research or study staff were directly involved in our day-to-day discussions. The first author and co-chairs of the HCV-PEG formed a subcommittee and have decided how to proceed with writing this paper.

The use of video “webinar-like” technology and the internet has enabled us to meet face to face and share documents in real time while living miles apart. The agreement to use videoconferencing technology saved a tremendous amount of time and money and has proven especially helpful since the start of the COVID-19 pandemic and social distancing protocols. The screen-sharing feature of videoconferencing enabled real-time editing of our documents and was incredibly helpful to the PEG to follow along during document edits. One instance of real-time document editing was utilized while creating and answering questions that we found pivotal in describing our experience as HCV-PEG members. Other edits to the main article were conducted live as well.

The PEG members wanted a more personal way to contribute besides reviewing documents and attending conference calls. By group consensus we asked each member to identify and submit three questions that they thought would be important to give an illustration of how and why we would be involved in a research project. The lead author and co-chairs pooled the submissions and comprised a questionnaire to be given to each member to complete. Each had individual answers and experiences for the questions, but had similarities as shown. We agreed that presenting a collective experience would better serve its purpose as shown in Table 3.

**Table 3:**
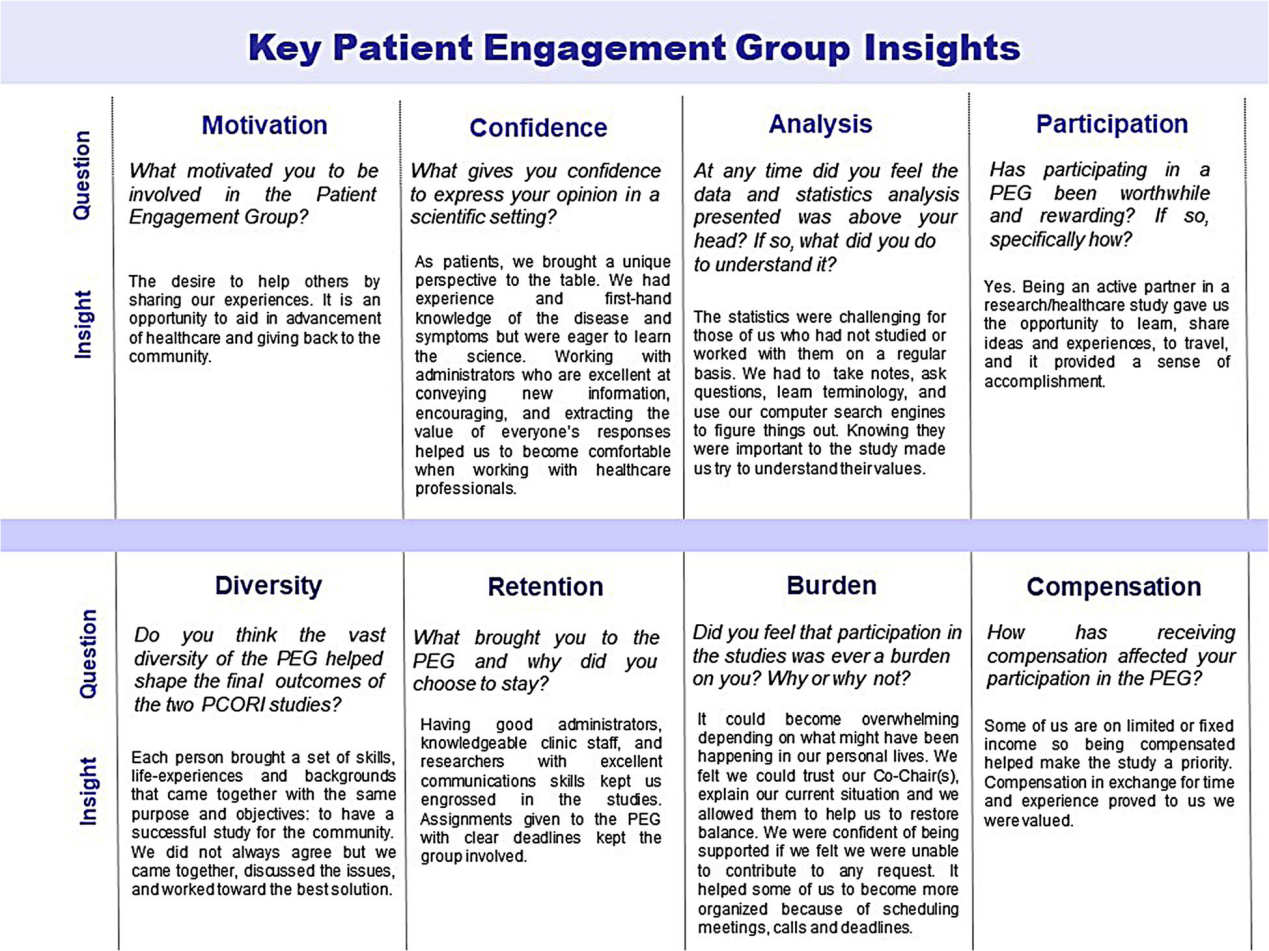
Key Patient Engagement Group Insights

## Conclusion

The UNC HCV-PEG’s focus has been on the treatment of chronic HCV including how patients respond to various drug regimens. Having patient input during all phases of drug development, testing and clinical trials has proven to be valuable not only for researchers but for the patients involved. In fact, it has proven to be so important that patient engagement is encouraged, and in some cases, mandated, by the US Food and Drug Administration (FDA) who is responsible for examining data on clinical benefits of drugs on patients’ health.

“The Prescription Drug User Fee Act (PDUFA) was a law passed by the United States Congress in 1992 which allowed the Food and Drug Administration (FDA) to collect fees from drug manufacturers to fund the new drug approval process … In order to continue collecting such fees, the FDA is required to meet certain performance benchmarks, primarily related to the speed of certain activities within the New Drug Application review process. The move towards imposing user fees to pay for the regulatory review of new medicines was the result of dissatisfaction among consumers, industry, and the FDA” [11].

Other patient engagement projects occur at the Center for Drug Evaluation and Research (CDER) within the FDA. The primary goal of patient-focused drug development is to better incorporate the patient’s voice in drug development and evaluation [12]. Some of these include:

- Facilitating and advancing the use of systematic approaches to collecting and utilizing robust and meaningful patient and caregiver input to more consistently inform drug development and regulatory decision-making.
- Encouraging identification and use of approaches and best practices to facilitate patient enrollment and minimizing the burden of patient participation in clinical trials.
- Enhancing understanding and appropriate use of methods to capture information on patient preferences and the potential acceptability of tradeoffs between treatment benefit and risk outcomes.
- Identifying the information that is most important to patients related to treatment benefits, risks, and burden, and how to best communicate the information to support their decision making [12].

Engaging patients in research and development in healthcare is becoming widely accepted because it makes research and its results more relevant for its patients [9]. Patient engagement groups like the UNC HCV-PEG who are included as active partners in research can provide useful input to many areas including how patients are treated during clinical trials, how they interact with research teams, and how the clinical benefits of drugs or devices are defined and evaluated. Further, patient researcher direct involvement in project operational and procedural decision-making can facilitate the development of new, relevant approaches to achieve project goals while maintaining the critical relevance to stakeholders. PCORI is highly invested in patient involvement as a means of understanding the informational needs of patients regarding healthcare treatment options and supporting research that helps patients and stakeholders make more informed healthcare decisions. PCORI “believes engagement influences research to be more patient-centered, useful, and trustworthy, and will ultimately lead to greater use and uptake of research results by the patient and broader healthcare community” [13]. Across the board with patient engagement, there have been reports of improved study conduct, including more effective recruitment, meeting enrollment targets, and substantial positive impacts on stakeholders, patients, and communities. There have been improvements to personal health and healthcare, increased skills and professional opportunities, and overall improvements to the relevance of research [13]. The FDA echoes the same sentiments as PCORI, requiring patient engagement in the evaluation of new drugs and devices on clinical outcomes [14].

The involvement of the UNC HCV-PEG has proven to be valuable to researchers but also to the actual members of the HCV-PEG. Experiences vary from person to person, but the consensus is that our group has contributed to the overall field of healthcare specifically in the area of DAA medications to treat chronic HCV. The HCV-PEG has felt valued by the doctors, statisticians, and academics who have interacted with the members during the studies. The lead investigators deserve the most credit for establishing and providing comprehensive agendas for the PEG so we could be focused on improving patients’ experiences during research participation. The investigators’ transparency and guidance has made the HCV-PEG’s works even more rewarding. Finally, the willingness of our studies’ sponsors to provide fair compensation for our time has been appreciated and made the group feel respected and recognized as valued members of the research team.

We believe our time and effort has been worth every minute during the past 7+ years. We feel that our experiences have given us opportunities to be representatives of positive change. We hope that the experiences presented in this article makes it easier for other patients and research teams to work collaboratively on research studies and to appreciate the patient input that brings a unique and valuable perspective.

## Data Availability

n/a

## Abbreviations

HCV: Hepatitis C
PEG: Patient Engagement Group
PCORI: Patient Centered Outcomes Research Institute
PCOR: Patient-Centered Outcomes Research
DAA: Direct Acting Antiviral
PDUFA: Prescription Drug User Fee Act
UNC: The University of North Carolina at Chapel Hill
UF: University of Florida
HIPAA: Health Insurance Portability and Accountability Act
PROP UP: Patient-Reported Outcomes Project
FDA: Food and Drug Administration
CDER: Center for Drug Evaluation and Research
